# Software tools for 3D nuclei segmentation and quantitative analysis in multicellular aggregates

**DOI:** 10.1016/j.csbj.2020.05.022

**Published:** 2020-06-03

**Authors:** Filippo Piccinini, Tamas Balassa, Antonella Carbonaro, Akos Diosdi, Timea Toth, Nikita Moshkov, Ervin A. Tasnadi, Peter Horvath

**Affiliations:** a Istituto Scientifico Romagnolo per lo Studio e la Cura dei Tumori (IRST) IRCCS, Cancer Research Hospital, Meldola, FC, Italy; b Synthetic and Systems Biology Unit, Biological Research Centre (BRC), Szeged, Hungary; c Department of Computer Science and Engineering, University of Bologna, Italy; d Doctoral School of Biology, University of Szeged, Hungary; e Doctoral School of Interdisciplinary Medicine, University of Szeged, Hungary; f National Research University Higher School of Economics, Moscow, Russia; g Doctoral School of Computer Science, University of Szeged, Hungary; h Institute for Molecular Medicine Finland, University of Helsinki, Helsinki, Finland; i Single-Cell Technologies Ltd., Szeged, Hungary

**Keywords:** Oncology, Microscopy, Cancer Spheroids, 3D Segmentation, Single-cell Analysis

## Abstract

Today, we are fully immersed into the era of 3D biology. It has been extensively demonstrated that 3D models: (*a*) better mimic the physiology of human tissues; (*b*) can effectively replace animal models; (*c*) often provide more reliable results than 2D ones. Accordingly, anti-cancer drug screenings and toxicology studies based on multicellular 3D biological models, the so-called “-oids” (e.g. spheroids, tumoroids, organoids), are blooming in the literature. However, the complex nature of these systems limit the manual quantitative analyses of single cells’ behaviour in the culture. Accordingly, the demand for advanced software tools that are able to perform phenotypic analysis is fundamental. In this work, we describe the freely accessible tools that are currently available for biologists and researchers interested in analysing the effects of drugs/treatments on 3D multicellular -oids at a single-cell resolution level. In addition, using publicly available nuclear stained datasets we quantitatively compare the segmentation performance of 9 specific tools.

## Graphical abstract

**Figure:**
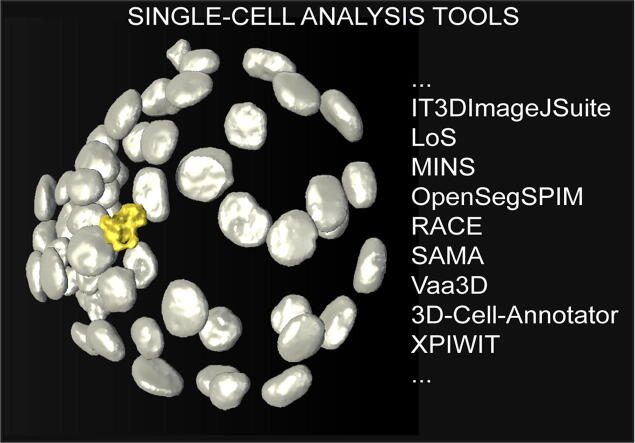

## 1 Introduction

The gap between standard *in vitro* cell cultures and complex *in vivo* models is getting tighter nowadays. Multicellular three-dimensional (3D) *in vitro* models, the so-called “-oids” (e.g. spheroids, tumoroids, organoids) have become widely known in the scientific community. They are currently used in numerous anti-cancer drug screenings and toxicology studies, and it has been extensively demonstrated that they are more reliable than classical flat (2D) cell cultures [1].

A geometry- and, consequently, functional complexity-based definition of various typologies of 3D multicellular models is reported in [2]. Briefly: *multicellular aggregate* is the general term for 3D cell–cell aggregates where there is no structural restriction; *spheroid* refers to a multicellular aggregate having a nearly spherical shape; *tumoroid* is a spheroid of cancer cells; *microtissue* is a 3D multicellular aggregate comprising more than one cell types that accomplish a specific function together; *organoid* indicates a self-renewing multicellular aggregate of irregular shape. In general, there is no globally dominant 3D model: all of them have limitations and opportunities. However, regardless of the experimental model, reaching single-cell resolution level in the analysis is fundamental to study the effects of molecular perturbations at a phenotypic level [3]. For instance, it is essential to see if there are populations of resistant cells capable of sustaining cancer regeneration [4]. Accordingly, the single-cell level behavioral analysis of 2D/3D cultures is the basis of most drug development and testing studies [5].

In 2018 we discussed the concerns, challenges and promises of 3D multicellular models [6]. We concluded that the complexity of -oids models strongly limits the manual analysis at the single cell level, and essentially require advanced software tools that are capable of performing 3D phenotypic measurements. In this work, we are focusing on the tools that help biologists in automating segmentation and analysis of cell nuclei in multicellular -oids. In particular, we have considered and tested the tools that are currently freely available for the scientific community. we should emphasize that segmenting nuclei in microscopy images is typically the first step of any quantitative analysis tasks. Numerous international competitions, including the ISBI Cell Tracking Challenge [7], [8] and the Kaggle 2018 Data Science Bowl [9], have challenged the bioinformatics community to further improve the available solutions.

To compare the different tools in real practical scenarios, we used two publicly available nuclear stained datasets related to a cancer spheroid and a mouse embryo, imaged with a light-sheet fluorescence microscope (LSFM) and a confocal microscope, respectively. The tools we tested were randomly assigned to 5 expert computer scientists working with microscopy images on a daily basis. We assessed the performance of each 3D segmentation tool, aiming to find the one that performs best using the Jaccard Index value obtained between the segmentation masks and the ground truth.

## 2 Freely available software tools for single cell analysis in 3D multicellular -oids

In this section we give a brief introduction into the main software tools that are currently freely available for segmenting and analysing single cells in multicellular -oids. The tools are presented in alphabetical order, and their main features are summarised in Tables 1 and 2.

**Table 1:** Tool features.

	IT3DImageJSuite	LoS	MINS	OpenSegSPIM	RACE	SAMA	Vaa3D	3D-Cell-Annotator	XPIWIT
	(version: 3.96)	(version: 1.0)	(version: 1.3)	(version: 1.1)	(version: 1.0)	(version: 1.0)	(version: v3.601)	(version: 1.0)	(version: 1.0)
*Documentation*									
User guide	X	X	X	X	X	X	X	X	X
Website	X	O	X	X	O	X	X	X	O
Video tutorial	O	O	O	X	O	O	X	X	O
Freely available tool	X	X	X	X	X	X	X	X	X
Open source code	X	X	X	X	X	X	X	X	X
Implementation language	Java	Mathematica/Java	MATLAB/C++	MATLAB	C++	Java/R	C/C++	C++	C++
Test dataset/demo	O	X	X	X	X	X	X	X	X

*Usability*									
No programming experience is required	X	X	X	X	X	X	X	X	O
User-friendly GUI	X	O	X	X	X	X	X	X	X
Intuitive visualization settings	X	O	X	O	O	X	X	X	O
No commercial licences are required	X	X	X	X	X	X	X	X	X
Portability on Win/Linux/Mac	Win/Linux/Mac	Win/Linux/Mac	Win	Win/Mac	Win/Linux/Mac	Win/Linux/Mac	Win/Linux/Mac	Win/Linux	Win/Linux/Mac

*Functionality*									
Automatic single-cell segmentation	X	X	X	X	X	X	X	O	X
Manual correction opportunity	O	O	O	X	O	O	O	X	O
Feature extraction	O	X	X	X	X	X	X	O	X
No human interaction is required	O	X	O	O	X	O	X	O	O

*Output*									
3D rendering	X	O	O	O	O	X	X	X	X
3D binary mask	X	X	X	X	X	X	X	X	X
Feature statistics	O	X	X	X	O	X	X	O	X

**X** available/yes; **O** not available/no.

**Table 2:** Tool references.

Tool	Link To Code/Executable	Main Scientific Reference	Average Yearly Citations*
IT3DImageJSuite	https://imagejdocu.tudor.lu/plugin/stacks/3d_ij_suite/start	Ollion et al. Bioinformatics 2013	36.5
LoS	www.physikalischebiologie.de/downloads	Mathew et al. BMC Bioinformatics 2015	5.3
MINS	http://katlab-tools.org	Lou et al. Stem Cell Reports 2014	9.7
OpenSegSPIM	opensegspim.weebly.com	Gole et al. Bioinformatics 2016	0.6
RACE	https://bitbucket.org/jstegmaier/race/downloads/	Stegmaier et al. Developmental Cell 2016	19.2
SAMA	https://montevil.theobio.org/en/content/sama	Paulose et al. PloS One 2016	2.2
Vaa3D	http://vaa3d.org	Peng et al. Nature Biotechnology 2010	49.3
3D-Cell-Annotator	www.3d-cell-annotator.org	Tasnadi et al. BioInformatics 2020	0.0
XPIWIT	https://bitbucket.org/jstegmaier/xpiwit/downloads/	Bartschat et al. BioInformatics 2016	4.6

*The analysis was performed on the 9th April 2020 using Google Scholar.

### 2.1 Iterative thresholding algorithm of the 3D ImageJ Suite (IT3DImageJSuite)

The *3D ImageJ Suite* of ImageJ/Fiji contains several algorithms for 3D segmentation. The 3D Iterative Thresholding (IT) tool is one of the most effective algorithms that may be used for 3D nuclei segmentation [10]. Regarding that using a single threshold level may not be enough to detect and extract the objects from an image, even if the threshold level is optimal, this IT algorithm tests all of the possible levels, and collects the objects yielded at different levels, fulfilling some *a priori* criteria defined by the user. These criteria include elongation, volume, shape and edges of the target object. Specifically, (*a*) the *elongation* criterion measures the object’s roundness, and uses the threshold level for the object where this roundness is maximal (i.e. the elongation is minimal); (*b*) the *volume* prior finds the threshold level where the volume of the object under extraction is maximal; (*c*) *shape* will try to find a threshold level that leads to minimal variation in the shape of the object upon adjusting the threshold levels; (*d*) when the *edges* criterion is set, the edges will be maximized upon seeking for a proper threshold level for an object. Since all possible threshold levels can be tested using IT, defining correct values for the mentioned criteria is essential when the tool is employed to segment single nuclei. The *3D ImageJ Suite* version 3.96 containing the IT algorithm we tested is available at: https://imagejdocu.tudor.lu/plugin/stacks/3d_ij_suite/start.

### 2.2 Lines-of-Sight decomposition (LoS)

Lines-of-Sight (LoS) is an automated method to segment 3D fluorescence images. The original concept of LoS was published by Asafi *et al*. [11] and was later adapted to a general robust cell/nuclei segmentation tool developed by Mathew *et al*. [12]. This method is based on the intrinsic definition of line-of-sight, which refers to the connecting line between two points on the surface within the inner volume of the shape. Therefore, when all the mutually visible surface points are in a line-of-sight position, the object is considered as convex shaped. In that concept, a single nucleus is expected to be convex, whilst touching nuclei are non-convex in general. This definition is the basis of the LoS approach that clusters the surface points and separates the borders of the nuclei. The whole pipeline has four main steps. It starts with a local intensity-based thresholding performed per slice. This first process separates the foreground and the background using an ImageJ plugin for automatic thresholding. The result is a binary image. In the second step, the segmented foreground components are extracted, and are stored together with their bounding box. Next, the algorithm determines the number of objects that can be calculated with the Euclidean distance transformation. Finally, the components are decomposed into approximately convex parts and an intensity-coded, multi-dimensional TIFF is provided as the output. The method is implemented in Mathematica and it is currently available at: https://www.physikalischebiologie.de/downloads.

### 2.3 Modular Interactive Nuclear Segmentation (MINS)

MINS stands for Modular Interactive Nuclear Segmentation [13], [14]. It is a MATLAB/C++-based segmentation tool tailored for fluorescent intensity measurements of 2D and 3D image data. MINS is a freely available tool widely used for segmenting single-cells in embryos, and it can be easily used for -oids as well. The MINS pipeline comprises three major cascaded modules: detection, segmentation, and cell position classification. Detection is based on a multiscale blob detection technique optimised for the localization of cell nuclei. Segmentation expands detection output to cover the full nuclear body. The developers chose Seeded Geodesic Image Segmentation (SGIS) as the base algorithm for this stage. Finally, classification is obtained through a clustering-based approach combined with robust shape-fitting that serves multiple purposes, including the separation of multiple embryos and removal of outliers, as well as the classification of inner and outer cells. The source-code and a Windows-only standalone executable of MINS version 1.3 are distributed at: http://katlab-tools.org.

### 2.4 OpenSegSPIM

OpenSegSPIM [15] is an open-source and user-friendly 3D automatic quantitative analysis tool for confocal/multiphoton/LSFM image data. OpenSegSPIM assumes no prior knowledge of image processing or programming and is designed to easily segment nuclei/cells and compute several features without requiring human interaction, except for setting a few initial parameters: an approximate nuclei diameter measurement and intensity adjustment of the image contrast. It allows to save the defined parameters and load them back for future analysis. OpenSegSPIM also includes: (*a*) a sub-cellular segmentation tool; (*b*) a simple post-processing tool for manually editing the segmentations obtained; and (*c*) an automatic batch process of time series and datasets. 3D masks and several object-based statistics are automatically provided as the output. The quality of the final segmentation of single-cells is negatively affected by the limits of thresholding in case of blurry objects and an improper separation of touching cells. The number of correctly detected objects strongly depends on the objects’ diameters. OpenSegSPIM is developed in MATLAB. Its source-code, standalone versions for Windows and Mac, documentation, user manuals and video tutorials are distributed at: http://opensegspim.weebly.com/. The currently available version of OpenSegSPIM is version 1.1.

### 2.5 Real-time Accurate Cell-shape Extractor (RACE)

Real-time Accurate Cell-shape Extractor (RACE) is an open-source analysis framework that works with large-scale images acquired with confocal microscope or LSFM [16]. It was designed to work as an automated 3D cell segmentation tool that takes advantage of state-of-the-art multi-core processors and graphics cards, hence it is capable of processing terabyte-sized datasets within 1–2 days. It provides an easy-to-use Graphical User Interface (GUI) for setting various parameters, including: input/output path of the images; type of the seeding points; version of the RACE algorithm to be used (e.g. Membrane, Nuclei or CSV seeding with ITK, NScale, CUDA or NScale + CUDA acceleration); microscope- and specimen-dependent parameters (e.g. the ratio of axial *versus* lateral voxel size in the image data, the minimum value of the radius range for iterative morphological closing, minimum seed size, maximum 2D segment size, cell volume boundary); and intensity-dependent parameters (e.g. binary threshold, H-maxima level, morphological watershed level). RACE requires single-channel 3D TIFF image stacks of fluorescently labeled cell membranes as an input. Additionally, it is possible to insert fluorescently labeled cell nuclei. The GUI offers the selection of initial seeding points for region growing-based segmentation to find cell boundaries. The user can import seeding points derived from external detection algorithms such as the Gaussian mixture models [17] or manually annotated seeds stored in the CATMAID database [18]. The user can select the type of seeding (membrane, nuclei or CSV) and computational acceleration options (ITK, NScale, CUDA or NScale + CUDA). Note that a CUDA-enabled device is required for the Graphic Processing Unit (GPU)-accelerated version of RACE. The source code, documentation and standalone versions (for Windows, Linux and Mac) of RACE are available at: https://bitbucket.org/jstegmaier/race.

### 2.6 Software for Automated Morphological Analysis (SAMA)

The Software for Automated Morphological Analysis (SAMA) is a Fiji plugin combined with a set of functions in R, an open-source program for statistical analysis [19]. SAMA aims to describe the morphological aspects of three-dimensional objects, as well as to reconstruct and analyze them with minimal human intervention and bias. Precisely, the user sets the measurements to be performed. The threshold value for the lumen analysis may be manually adjusted. SAMA supports any image file format supported directly by ImageJ/Fiji. The plugin operates with two main components: SAMA-images and SAMA-analyze. SAMA-images produces quantitative data for every image stack in 3 tiers. In Tier 1 the user can analyze basic morphometrics (3D structures; shape, volume and position parameters), complexity and lumen morphology either simultaneously or individually. In Tier 2 the user manually sets the threshold for lumen analysis and in Tier 3 lumena are identified. SAMA-analyze operates the part of SAMA written in R. It gathers the output of sama-images and enables to represent, analyze and export these output data. The website of SAMA (https://montevil.theobio.org/en/content/sama) contains the Fiji plugin (compatible with Windows, Mac and Unix), the source code and a detailed technical description of the software, including sample images.

### 2.7 3D Visualization-Assisted Analysis software suite (Vaa3D)

3D Visualization-Assisted Analysis (Vaa3D, previously known as V3D) is an open source platform for large-scale bioimage visualization, analysis and management of multidimensional microscopic images [20]. Vaa3D has a rich set of functions and plugins, and it has been used in several bioimage informatics applications. It runs locally as a stand-alone application on Mac, Linux and Windows machines. Similarly to ImageJ, Vaa3D is extensible through a plugin architecture. The currently available version of Vaa3D is version 3.601. The cell segmentation toolkit is distributed as a plugin of the Vaa3D system. It is a user-friendly, fully-automatic tool requiring just a few parameters: the number of diffusion iterations, the fusion threshold, and the minimum region size. The tool performs the segmentation only on 8-bit files. The algorithm uses a gradient vector flow based segmentation [21]. The tool determines the total number of cells and can be used to measure the volume of the cells. The segmented image is saved as a v3draw file format, which is easily convertible to a standard TIFF format by using ImageJ. The Vaa3D software suite, as well as source-code, documentation, user manuals, testing data and video tutorials are distributed at: http://vaa3d.org.

### 2.8 3D-Cell-Annotator

3D-Cell-Annotator [22] is a patch for the segmentation plugin of the Medical Imaging Interaction Toolkit version 2018.04 (MITK) [23] written in C++/CUDA and released as a full MITK distribution. It allows to segment single cells and nuclei in 3D, starting from a 3D dataset typically acquired with confocal, multi-photon or LSF microscopes. It uses 3D active contours with shape descriptors as prior information for true single cell annotation in a semi-automatic fashion. A label for each object is provided to start contour evolution. Annotation can be provided cell-by-cell manually or semi-automatically by placing initial seedpoints. While the general active surface algorithm may output clusters of objects when multiple cells share boundaries, the proposed selective active surface applies forces to fulfil shape descriptor values provided by the user [24]. Two such descriptors are used: sphericity and the volume of the object. These prior parameters can be fine-adjusted with high precision during surface evolution to obtain segmentation at a single cell level. The obtained segmentations are automatically exported as 3D masks. However, no object-based statistics are automatically computed as an output. 3D-Cell-Annotator works on Windows and Linux operating systems, but not on Macintosh. It requires a CUDA-enabled GPU and a recent version of NVidia driver. The 3D-Cell-Annotator enabled MITK distribution, the source code, documentation, video tutorials, and test datasets can be downloaded at: www.3D-cell-annotator.org. The currently available version of 3D-Cell-Annotator is version 1.0.

### 2.9 XPIWIT

XPIWIT [25] stands for XML Pipeline Wizard for ITK. It is an XML-based wrapper application for the Insight Toolkit (ITK) that combines the performance of a pure C++ implementation with a graphical setup of dynamic image analysis pipelines. One of the most promising algorithms for nuclei segmentation included in XPIWIT is the *Threshold of Weighted intensity And seed-Normal Gradient dot product image* (TWANG) segmentation [26]. The current version of XPIWIT (i.e. version 1.0) incorporates about 70 different ITK filters, and can be extended with new functionalities using the provided template files that facilitate the implementation of new modules. To apply a predefined XML processing pipeline on an image dataset automatically, XPIWIT can also be executed from the command prompt with command line input arguments. Alternatively, a configuration text file can be piped to the executable. This configuration file needs to contain the output path, one or more input paths, the path of the XML file describing the pipeline, and may be customized using further optional parameters as described in the provided documentation. XPIWIT also has a useful GUI that allows the user to drag and drop the algorithms available in the ITK to the stage, and couple them to form an image processing pipeline. XPIWIT has successfully been applied to segment and visualize terabyte-scale 3D+ time LSFM images of developing embryos. Its source-code, documentation and standalone versions are available at: https://bitbucket.org/jstegmaier/xpiwit/downloads/.

## 3 Further freely available tools

In this section we discuss several other tools for 3D segmentation/analysis. These were not tested in the current work for different reasons: (*a*) In group I we report those tools that have no 3D splitting algorithms implemented. Accordingly, they can be used to segment only isolated 3D objects. (*b*) In group II we briefly describe several tools that were employed in scientific works in the past years, but are not available/supported anymore. (*c*) There are also several tools (group III) that require multiple stainings. Accordingly, they are not designed for the datasets used in this testbed, containing only the nuclear signal. (*d*) Finally, we list 3D deep-learning tools (group IV). Despite the great interest and expectation of the community for these tools, there is no standard pre-trained model available for single cell segmentation.

### 3.1 Group I: Tools without object splitting algorithms

#### 3.1.1 Ilastik

Ilastik is one of the most well-known tools for segmenting the background and foreground in 2D and 3D images. It is based on a pixel classification and it allows the user to easily define several classes of interest by manually annotating representative objects. Using supervised pixel-level classifiers, it segments the image [27]. A prestigious scientific review of this tool has been published recently [28]. Most likely, the reason for Ilastik’s having become so popular is its user-friendly and well documented nature. Thanks to a very intuitive GUI, it does not require any programming skills, and allows even users without expertise in image processing to perform segmentation and classification. Despite its wide usability, Ilastik is not able to segment the single cells in the datasets considered in this work, because no 3D splitting algorithms have been implemented until now. Accordingly, all the touching nuclei are considered as a single object. Ilastik is available at: www.ilastik.org.

#### 3.1.2 Markov Random Fields 3D (MRF3D)

Robinson *et al*. developed a 3D segmentation tool based on Markov Random Fields (MRF3D) [29]. In their representation, vertices of the Markov model represent pixels in digital images of cells, and edges represent spatial relations between the variables in a non-tree Markov Random Field. Modelling is carried out for each pixel and its four immediate neighbours (above, below, left and right) in the digital image. The developers applied the tool on 3D image stacks of organotypic 3D cell culture models comprising prostate cancer cells co-cultured with cancer associated fibroblasts (CAFs). The tool does not provide the opportunity to split touching objects. Accordingly, it can not be successfully used to segment our datasets containing touching nuclei. MRF3D is a tool developed in MATLAB. It has no GUI. The source-code, description and data files are distributed as Supplementary Material of the original publication [29].

### 3.2 Group II: Unsupported or currently unavailable tools

#### 3.2.1 CellFrost

CellFrost [30] is a fully automated tool for segmenting 2D and 3D cellular time-lapse data. The authors proposed a cellular shape tracking system that involves two main steps. First, a coherence-enhancing diffusion filter is applied to each frame in order to reduce noise. Then, as the second step, a cell boundary detection is executed by minimizing the Chan-Vese model [31] in two different (fast level set-like and graph cut) frameworks. This tool was developed in MATLAB and has no graphical user interface. CellFrost is an easy-to-use software that requires only 10 parameters: (1, 2) the path to the input/output folder; (3, 4) the number of slices of each frame, and the number of frames of the analyzed sequence; (5, 6, 7) the weights for the curvature term and the foreground and background fidelity terms of the Chan-Vese model; (8) the maximum number of iterations to be performed; (9) minimum object size; (10) the diameter of the overlap. Originally it was developed for applications on time-lapse data, but with a proper naming convention it can process non-time-lapse data too. Unfortunately, the website cannot be reached any more, thus the tool can be obtained from the developers only.

#### 3.2.2 CellSegmentation3D

CellSegmentation3D is one of the first fully-automated, freely-available tools that appeared in the literature for the segmentation of cell nuclei in 3D microscopic images [32]. It was specifically designed to segment closely juxtaposed or touching nuclei in images. The segmentation approach includes three stages: (*a*) a gradient diffusion procedure; (*b*) gradient flow tracking and grouping; (*c*) local adaptive thresholding. The tool was validated on images of *C. elegans* and zebrafish cells for nuclei segmentation. CellSegmentation3D was developed in C/C++. It has a command line interface, and it works with *3D Analyze format* only. The source code and the authors’ original files are distributed as Supplementary Material of the original publication [32]. Unfortunately, the tool is not supported any more.

#### 3.2.3 FARSIGHT

FARSIGHT is a tool originally developed to delineate and classify key structures in images of 3D brain tissue samples. It has become a very popular tool for analysing 3D images in general according to the systematic “divide and conquer” methodology [33]. In the implemented approach, first the image is split into different channels. Then, distinct automated 3D segmentations are applied on the various channels to delineate different parts of interest. After an application of image-based measurements, features are extracted for each object. Based on these features a classification is done for the different phenotypes. For the whole process, no human interaction is needed: the tool is fully automated. Furthermore, no commercial licence is required, and it is released for multiple operating systems (i.e. Windows, Linux, MAC). The software was originally available at: www.farsight-toolkit.org. Unfortunately, today the website is offline and the tool cannot be reached through the official hyperlink.

#### 3.2.4 Pipeline for Automated oR interactive SegMentation of Images (Parismi)

Pipeline for Automated oR interactive SegMentation of Images (Parismi) is another tool that can be used to segment nuclei in 3D [34]. The tool is designed as an ImageJ distribution equipped with the plugin that implements the software, and it is available under the Plugins menu as “A0PipelineManager”. The user first opens the target image in ImageJ, and can define the segmentation pipeline using the GUI of the plugin by selecting the algorithms from a list. The segmentation process is executed according to a two-step method. First, the user has to define the seedpoints for the nuclei, either manually by using the embedded GUI, or by using a machine learning algorithm available in the software. The algorithm is similar to Ilastik [27], but it is trained to detect the seedpoints instead of a semantic segmentation. The machine learning model can be trained in a semi-supervised fashion by reviewing the proposed nuclei and fixing the detections using the same GUI. After this step, the model can be re-trained, and this iterative process can be further continued until the desired accuracy is reached. As a next step, a 3D active contour approach is used for segmentation. The contour grows from the detected initial seeding points, and it stops when it reaches the object’s boundary, and a usual curvature-based smoothness term is applied. The software can theoretically run on any platform, but the required libraries are precompiled to the OS X platform only, and runs properly with Java version 1.8 or less.

### 3.3 Group III: 3D single-cell analysis tools incompatible with the test environment

#### 3.3.1 Biologically Constrained optimization based cell Membrane Segmentation (BCOMS)

Biologically Constrained Optimization based cell Membrane Segmentation (BCOMS) [35] is a tool developed to automate an accurate extraction of cell shapes in *C. elegans* embryos. Compared to typical iteration-based approaches, BCOMS first computes an automatic segmentation with different parameter sets, and then the optimal segmentation is selected by an evaluation. In this evaluation step, the objective task measures the correlation between the membrane image and the segmentation results. Segmentation is achieved by a two-step framework including (*a*) embryonic region segmentation using a level set method, and (*b*) cell membrane segmentation using a segmented nuclei-seeded watershed. The software was developed in MATLAB, and is applicable on 4D (3D – time-lapse) data only. BCOMS is well documented and its usage is very easy to learn. Its source code, user-guide and sample data are available at: https://github.com/bcomsCelegans/BCOMS. However, BCOMS is not directly applicable on nuclear segmentation. It was developed for segmenting cell membrane. Accordingly, we did not test it on the datasets considered in this work.

#### 3.3.2 CellDissect

CellDissect [36] is a MATLAB based tool for automatic 2D cellular and 3D nuclear segmentation of image data. Besides its easy use, software, a step-by-step user guide is available to help understanding the workflow. No programming skills are required for the proper usage of CellDissect. However, before segmentation, four group of parameters are recommended to be set up. In group 1, the user can specify general features, such as the operating system used. Also, the images under processing can be made visible on an optional basis. In group 2, parameters for the data are required, such as input and output directories, and information on the channels to be considered. Group 3 of the parameters are designed to help the user to modify the data visualization (e.g. change brightness, show or hide cell and nuclear boundaries). Finally, in group 4, parameters for the objects can be set, such as the minimum/maximum size of the cells/nuclei and the lowest/highest slice that still contains an object. Nevertheless, the developers also provide pre-set parameter files that can be loaded into the application. The software’s method is based on an adaptive thresholding approach. Binary images are generated from maximum intensity projections of DAPI images containing information about the nuclei, where the objects are filtered based on the previously defined parameters. In the next step, the different *z*-layers are analysed to separate connected nuclei, while trying to preserve the nucleus size and keeping the highest number of individual objects. In the last steps, a watershed algorithm labels the individual cells, and the boundary of the nucleus is determined based on pixel intensities. The source code, documentation, and sample images are publicly available at: https://www.dropbox.com/sh/egb27tsgk6fpixf/AADaJ8DSjab_c0gU7N7ZF0Zba?dl=0. CellDissect works with 3D datasets, but only on those related to monolayer cell cultures. Without home-made code arranging, CellDissect can not be used to analyse multicellular spheroids and embryos comprising several layers of cells.

#### 3.3.3 LimeSeg

Machado *et al*. have recently released LimeSeg, a surface segmentation and reconstruction tool implemented as a 3D ImageJ/Fiji plugin [37]. It is a particle-based active contour method that can segment non-overlapping objects in 3D images. It is a semi-automatic tool having tested for segmenting lipid membrane in several scenarios. The most important parameters to provide are: (*a*) the equilibrium spacing between the particles used by LimeSeg, required to delineate the 3D shape; (*b*) the pressure exerted on the surface; a positive value will lead to expansion, while a negative value will lead to shrinkage; (*c*) Z-Scale; (*d*) the size over which each particle will look for a local maximum; (*e*) the number of optimization steps. The source code, video tutorials, user manuals, image datasets and script examples are distributed at: https://imagej.net/LimeSeg. LimeSeg produces segmentations in ply format: ply is a format for storing graphical objects described as a collection of polygons. Converting ply files into a 3D label-map (e.g. a multi-dimensional TIFF with unique ID for each segmented object) is not straightforward, and there is no direct way to execute it in LimeSeg. Accordingly, it is impossible to quantitatively compare the obtained masks with those generated by the other tools tested in this work.

## 3.3.4 PArameter-free Generic Iterative Thresholding Algorithm (PAGITA)

PAGITA [38] is an automatic algorithm to classify and simultaneously segment cells/nuclei in 3D/4D images. Segmentation relies on training samples that are provided by the user through an iterative thresholding process. This algorithm can segment cells/nuclei even when they are touching, and remains effective under temporal and spatial variations of intensity. However, its usage may be time-consuming due to the threshold values to be tested. It can be considered as a more complex classification-based algorithm arising from the *Iterative Thresholding* approach implemented in the *3D ImageJ Suite* (https://imagejdocu.list.lu/plugin/stacks/3d_ij_suite/start) [10]. The main idea of the algorithm is that it uses machine learning to improve the results of segmentation. The learning step is an interactive process. The user first selects representative cells/nuclei of different phenotypes by clicking on their locations inside the 3D or 4D dataset. The objects at the clicked positions are then segmented through an iterative thresholding procedure using the user-supplied volume estimates as a reference. Next, the user validates the proposed nuclei segmentation, and finally 3D descriptors are computed from this set of validated nuclei. The main joint segmentation/classification procedure is subsequently applied to all time-points. PAGITA has been implemented as an open-source plug-in for ImageJ and is publicly available for download along with a tutorial and sample data at: http://imagejdocu.tudor.lu.

### 3.3.5 Repulsive parallel Hill-Climbing (RPHC), RoiEdit3D, and SPF-CellTracker

The Repulsive Parallel Hill-Climbing (RPHC) algorithm is an optimization method to analyze cells in four dimensions of space and time [39]. It was designed as a multi-object tracking solution, and uses a numerical optimization-based peak detection technique for object detection. Neither its source code, nor a standalone version is available. RoiEdit3D and SPF-CellTracker are created by the same group of Authors as RPHC, but they use different approaches in these [40], [41]. The main task of SPF-CellTracker is to detect and track cells in time-lapse 3D image series, and it is especially intended for 4D live-cell imaging of *C. elegans*. The source code of SPF-CellTracker is available at the following github repository: https://github.com/ohirose/spf. RoiEdit3D is a graphical user interface for visualizing and correcting tracking results. It is based on ImageJ/Fiji in MATLAB through Miji [42], and is available at: https://dx.doi.org/10.6084/m9.figshare.3184546. The publicly released-versions of these tools can be used to export tracking data, but they have no built-in functions to export the results of the 3D cell-detection as masks. Therefore, we could not compare the performance of these tools to that of other software solutions, however, we mention them because they perform object segmentation without requiring any external software tools.

### 3.3.6 3D Membrane Morphological Segmentation (3DMMS)

3D Membrane Morphological Segmentation (3DMMS) is a MATLAB plugin designed to segment and analyse embryonic morphological features [43]. In particular, 3DMMS concentrates on segmenting membrane images at the single-cell level. It requires two different images, one for the nuclei signals, and one for the membrane staining. First, a statistical intensity normalization is applied to improve image quality. Then, the membrane signal is enhanced by a Hessian transformation. After this membrane signal enhancement, the boundary is binarized. The regions are analysed after thresholding, using a custom algorithm based on PCA. A surface regression is applied on the result to achieve a complete surface without any missing points. After the algorithm reconstructs the surface completely, a combination of membrane and nuclei-based segmentation is performed to yield the final result. 3DMMS’s source code is available at: https://github.com/cao13jf/3DMMS_new. 3DMMS data and images are available at: https://figshare.com/articles/Dataset_for_MMS/7781777/1. However, the application of 3DMMS is restricted to 3D datasets with both membrane and nuclei signals. Accordingly, it is not applicable to the two nuclear datasets considered in this work.

### 3.4 Group IV: Deep-learning tools for 3D segmentation

#### 3.4.1 CDeep3M

CDeep3M [44] is a cloud-based convolutional neural network (CNN) solution for image segmentation. It is capable of working on both large and complex 2D and 3D image data derived from different types of microscopes (e.g. electron, light and X-ray). The developers of CDeep3M have modified a CNN network to make it applicable to a wide variety of segmentation tasks, such as object segmentation. To solve difficult tasks and process large datasets, the image volumes are automatically split into smaller pieces with overlap, then augmented, processed and trained on a graphical processing unit (GPU) in a parallel way. A notable module offered within the tool can apply transfer learning on the pipeline. This technique helps the user to re-use and refine a previously trained neural network. By applying transfer learning on previous models, it is claimed that one can save up to 90% training time for the custom dataset. The authors utilise Amazon Web Services for their software. This is an online platform offering a place for web-tools. Online availability has its benefit: it is convenient for the end-users to have a ready-to-use tool with minimal hardware/software setup and maintenance requirements, but as a downside it has usage fees. However, the authors provide the tool freely in a docker version (or an installable package for advanced users), but in that case hardware requirements appear. The software is available at: https://github.com/CRBS/cdeep3m, and several trained models are collected at: http://cellimagelibrary.org/cdeep3m. The prediction is a probability map of foreground and background. Accordingly, currently CDeep3M is a semantic segmentation tool that cannot be considered for object separation. Notably, CDeep3M’s github page also contains some suggestions regarding how to post-process the data in order to obtain 3D single-cell segmentations and execute morphological analysis tasks.

#### 3.4.2 QCA Net

Quantitative Criterion Acquisition Network (QCA Net) [45] is a CNN-based instance segmentation algorithm to segment nuclei and label the detected objects. The input of QCA Net is a time-series of 3D fluorescence microscopic images and the output is an instance segmentation image at each time point. QCA Net can extract several statistics, such as the time-series data for the nuclear number, volume, surface area, and center of gravity coordinates. QCA Net was originally designed to segment the nuclei of mouse embryos, but it can be easily applied also to -oids. QCA Net consists of two subnetworks called Nuclear Segmentation Network (NSN) and Nuclear Detection Network (NDN). As the names of these networks suggest, NSN is used for nuclear segmentation, while NDN for nuclear identification. The main aim of QCA Net is performing instance segmentation of the images at each time point so that: (*a*) Input images are pre-processed for normalising the intensity values of the pixels to prevent value divergence and gradient disappearance in learning; (*b*) Next, NSN performs semantic segmentation of the nuclear region; (*c*) In parallel, NDN performs semantic segmentation of the center of nuclei; (*d*) Finally, the nuclear region estimated by NSN is segmented by marker-based watershed from the center identified by NDN. QCA Net is an open source tool implemented in Python. The source code is available at: https://github.com/funalab/QCANet.

#### 3.4.3 StarDist

StarDist [46] is a deep learning approach for 2D and 3D object detection and segmentation. In their most recent application, Weigert *et al*. have adopted and extended their previous work [47], in which they proposed a method for cell nuclei localization with star-convex polygons on 2D image based data. Their improvement has extended the original approach to 3D by exchanging polygons for polyhedras. To predict the star-convex polyhedron representation, as well as the probability that a pixel is part of an object, a convolutional neural network is used. More precisely, the network is trained to predict the distance between each pixel and the object’s boundary, together with object probability, specified as the distance from the nearest background pixel. For this recently released 3D extension of StarDist the developers have made optimizations to reduce the required computational resources, although the training time can be relatively long, alike in case of other deep learning methods. The system was developed in python, using Tensorflow. A user-guide on how to prepare the training data, as well as guidelines and sources are available at: https://github.com/mpicbg-csbd/stardist. We were unable to test StarDist with the two datasets considered in this work, because there is no appropriate pre-trained model and we did not have sufficient annotated data for building a custom model.

#### 3.4.4 3D U-Net

In contrast to the StarDist method, 3D U-Net is a generalization of the basic bottom-up style U-Net network [48]. A general idea of the 3D U-Net network is to separate the foreground from the background by predicting the nuclei/background label probabilities for each pixel, followed by selecting the class with the highest probability to achieve the semantic segmentation. In case the class label of the voxels is nearly correct in the predicted image, then the instances can be extracted easily by applying a connected components algorithm. However, this method may lead to suboptimal solutions when multiple nuclei are touching in the predicted image. For this reason, the authors have proposed a weighted loss function that forces the optimization algorithm to focus on the important points where the cells are touching. In case the separating regions between cells are labeled as background or as a third class and applying connected component search may lead to a correct instance segmentation. 3D U-Net has no appropriate pre-trained model for segmenting the single-cells of the two datasets considered in this work.

#### 3.4.5 Zhao et al. 2018 approach

Zhao *et al*. [49] proposed a two-step deep learning approach for segmenting 3D data. The method combines detection with a sparse annotation strategy for training a deep learning 3D model, without requiring extensive full voxel annotation. 3D deep learning models typically face a critical challenge: the insufficient availability of training data due to various difficulties related to annotation. A full voxel annotation strategy incurs high workloads and costs because only experts can annotate biomedical images properly, and currently no direct annotation technique is available for 3D biomedical images. Accordingly, in most cases the set of available labelled objects is too small for an efficient training of deep learning models, which is the major obstacle limiting further development of these models for 3D segmentation. The training proposed by Zhao *et al*. consists of two major components: (*a*) 3D bounding boxes for all objects in the dataset and (*b*) full voxel annotation just for a small fraction of the objects. Labeling a 3D bounding box for each instance is about 30 times faster than labeling a 3D bounding box of the cell. The proposed approach was tested on several biomedical image dataset. These experimental results indicate that with full annotated boxes and a small amount of masks, this approach performs similar to other methods based on full annotations, however the time required for the former one is significantly shorter. Unfortunately, the code is not available, therefore we could not test this approach.

## 5 4 Commercial tools

In this work, we have considered only the freely available tools for 3D segmentation and analysis of single nuclei in multicellular -oids. However, it is very important to mention that several commercial solutions are also available, and offer a great and wide variety of services. For instance, IMARIS (Bitplane, headquarters: Belfast, UK, https://imaris.oxinst.com) provides a large set of segmentation and analysis solutions for 3D datasets. The software is well documented and versatile. A trial version of the full software is available for a limited time, but a freely available 3D viewer has also been released recently. AVIRIS (arivis AG, headquarters: Munich, Germany, https://www.arivis.com) is another full-featured solution. The website recommends numerous video tutorials, and in particular, the “*3D Magic Wand*” tool allows the user to segment even complex objects in 3D with just one click. Similar to the previous ones, AMIRA (Thermo Fisher Scientific, headquarters: Waltham, Massachusetts, USA, https://www.thermofisher.com) and VOLOCITY (Quorum Technologies Inc, Laughton, UK, https://www.quorumtech.com) are 2D-5D visualization and analysis tools, providing a wide range of options for segmenting/analysing single cells in multicellular -oids and embryo datasets like those considered in this work.

## 6 5 General purpose image analysis software

The most well-known free and open source image analysis tools for 3D segmentation and analysis are BioImageXD [50], Cell Profiler 3.0 [51], Fiji [52], ICY [53], ImageJ [54], MITK [23] and Slicer 3D [55]. The reason why we have excluded these tools from our quantitative comparison is that there is no “official/validated” pipeline/approach to use them for segmenting single cells in multicellular -oids and embryo datasets. Basically, these tools provide a number of image processing steps, and expert users may incorporate them into other 3D segmentation workflows. However, this way the specific pipeline/approach used becomes user-dependent. Accordingly, we have decided to exclude these general image analysis tools from quantitative comparison because there is no unique way for their use.

## 7 6 Tool comparison

In order to compare the freely available tools for 3D segmentation of the single nuclei in 3D datasets, we used the two publicly available 3D datasets considered in [22] as a testbed.

The first one is a multicellular spheroid composed of 52 cells with stained nuclei, imaged with an LSFM. It was originally used by [15] and it is publicly available under the name of “*Neurosphere_Dataset*” at: http://opensegspim.weebly.com/download.html. Accordingly, hereafter we refer to this dataset as *Neurosphere*. The second one is a mouse embryo dataset (hereafter referred to as *Embryo*), composed of 56 cells imaged with a confocal microscope. It was originally used by [14]. It is named: “*4May15FGFRionCD1_SU54_LM1*”, and it is publicly available in the native microscopy format (i.e. Zeiss “.lsm” format) at: https://figshare.com/articles/Raw_images_for_Saiz_et_al_2016/3767976/1.

In our analysis, we focused on the nuclear staining channel only.

**Fig. 1:**
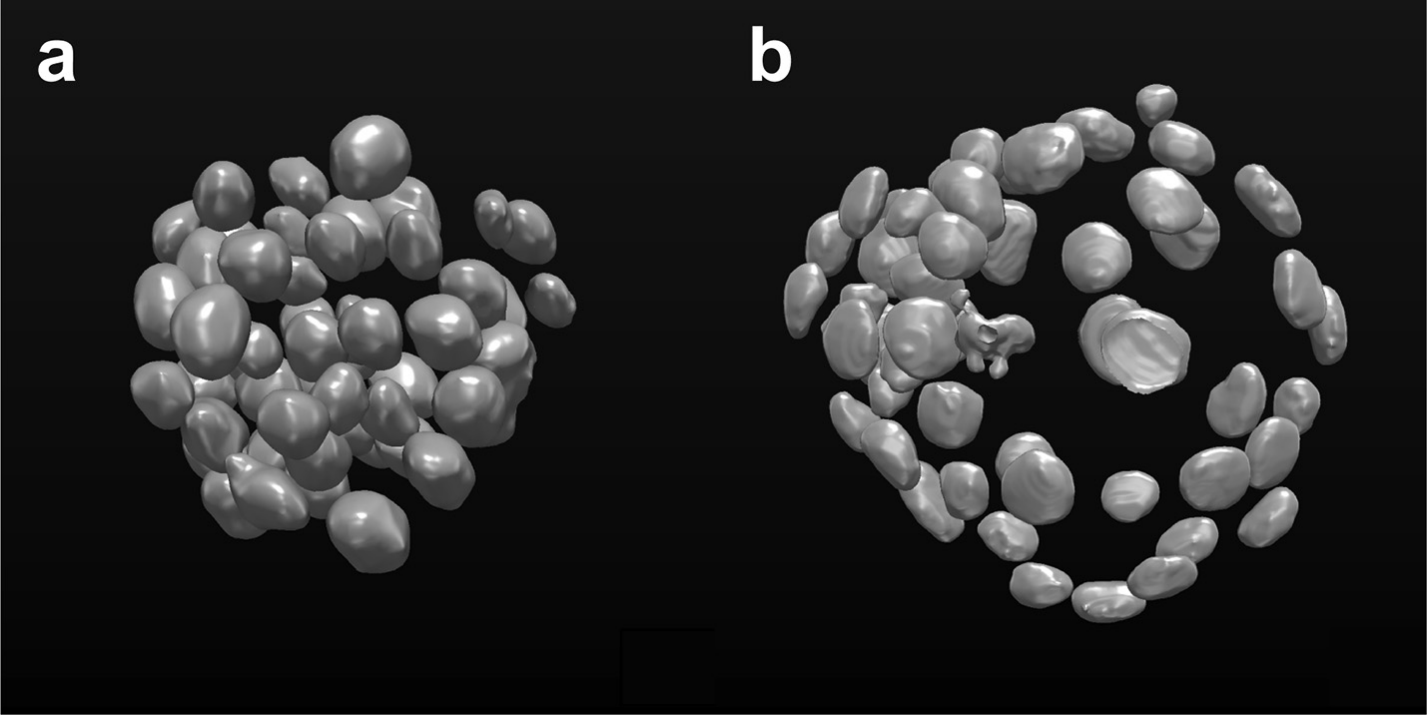
3D representation of the ground truth segmentations of single nuclei in the (a) multicellular spheroid and (b) mouse embryo datasets considered as the testbed of this work. Images were created by using MITK.

For both of the datasets, the ground truth (GT) was created by an expert microscopist by manually segmenting single nuclei using MITK. Fig. 1 reports a 3D representation of the ground truth segmentations of each single cell in both datasets.

In order to quantitatively compare the tools, we have randomly assigned them to 5 bioimaging expert computer scientists, asking them to use the tools for segmenting the single nuclei of the two proposed datasets, aiming at obtaining the best 3D segmentation possible. It is worth noting that the computer scientists involved in the experiments are expert researchers who work with microscopy images on a daily basis. They had no GT, nor training data set at their disposal, but however, no time constraints were defined for the test. For analysing the proposed datasets and obtaining the masks with the automatic tools, each operator required no more than 10 min, whilst testing the semi-automatic tool (i.e. 3D-Cell-Annotator) required a few hours. The experts were just demanded to avoid creating scripts to automatically analyse the segmentation parameters. In several cases, they also contacted the authors of the tools to ask for suggestions regarding software use (see Acknowledgements). Practically, these experts simulate a trained computer scientist researcher with bioimage analysis background interested in using the tool with an opportunity to exploit the available material and eventually contact its developers. Finally, one additional operator collected the masks and computed the 3D Jaccard Index (JI) values.

JI, also known as Intersection over Union (IoU) or the Jaccard Similarity Coefficient, is a well known metric used for evaluating the similarity of two sample sets (e.g. *A* and *B*). *JI(A, B)* is mathematically defined as the size of the intersection (i.e. |*A* ∩ *B|,* the number of overlapping voxels) divided by the size of the union (i.e. |*A ∪ B|*) of the sample sets, according to Eq. (1):

(1) $$JI\left(A,\;B\right)=\left|A\cap B|/|A\cup B\right|=|A\cap B|/\left(\left|A\right|+\left|B|-\;\right|A\cap B|\right)$$

The MATLAB code for computing the JI for 3D intensity-coded masks for multiple objects (i.e. masks with a unique ID for each object) is provided at: www.3d-cell-annotator.org/download.html (Supplementary Material 1 file - “SM1”). In the provided implementation, the 3D JI calculation method pairs the objects in the GT (i.e. the mask of objects created by the expert microscopist manually segmenting single nuclei) with the object of the predicted masks having maximum overlap, assigning a zero value to objects with less than 50% of overlap with any of the predicted masks. Then, for each GT object, it computes the JI Finally, the 3D JI is calculated by averaging the JI values obtained for the single objects.

**Fig. 2:**
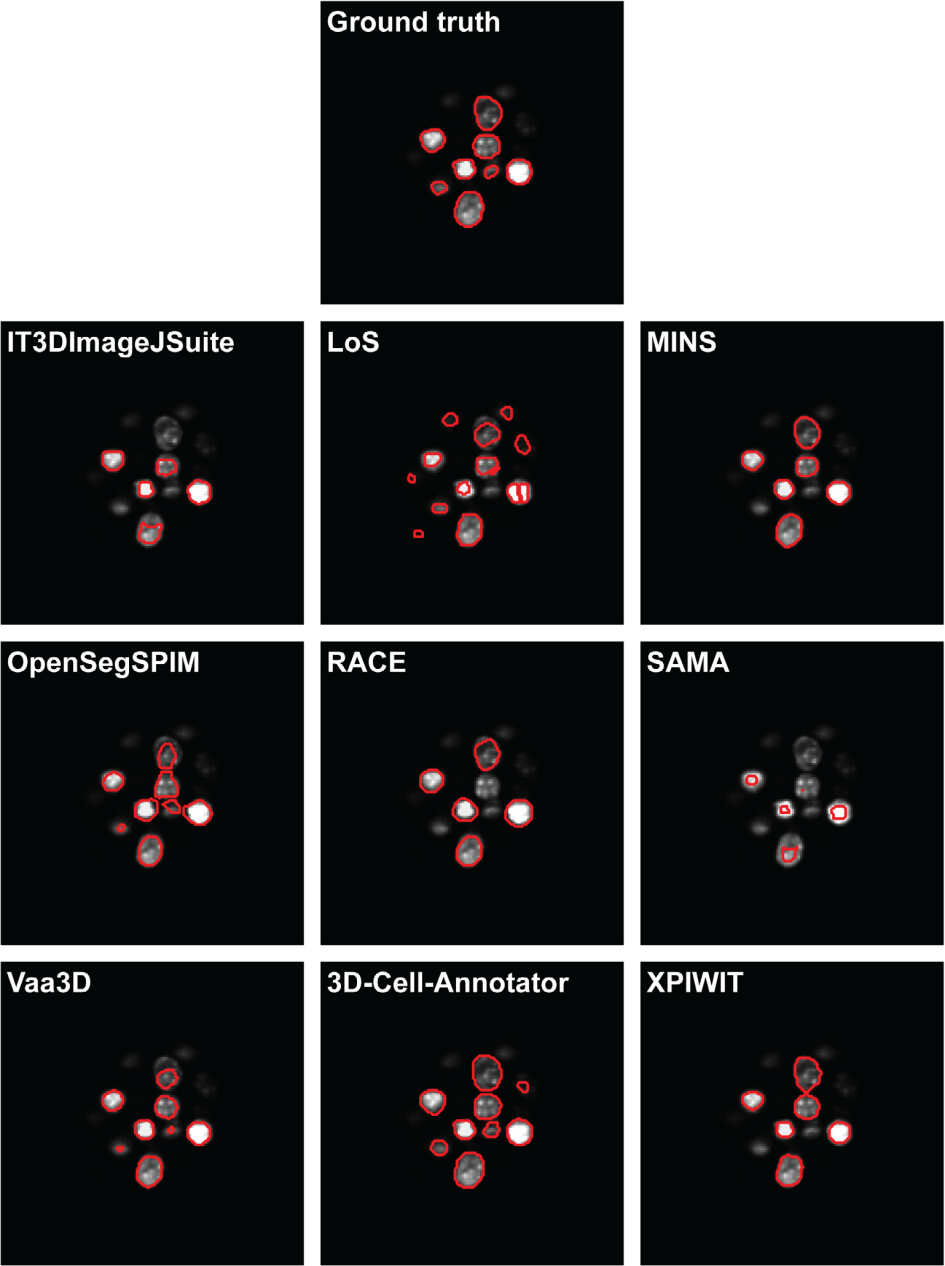
A representative section of the segmentation masks obtained on the Neurosphere dataset.

**Fig. 3:**
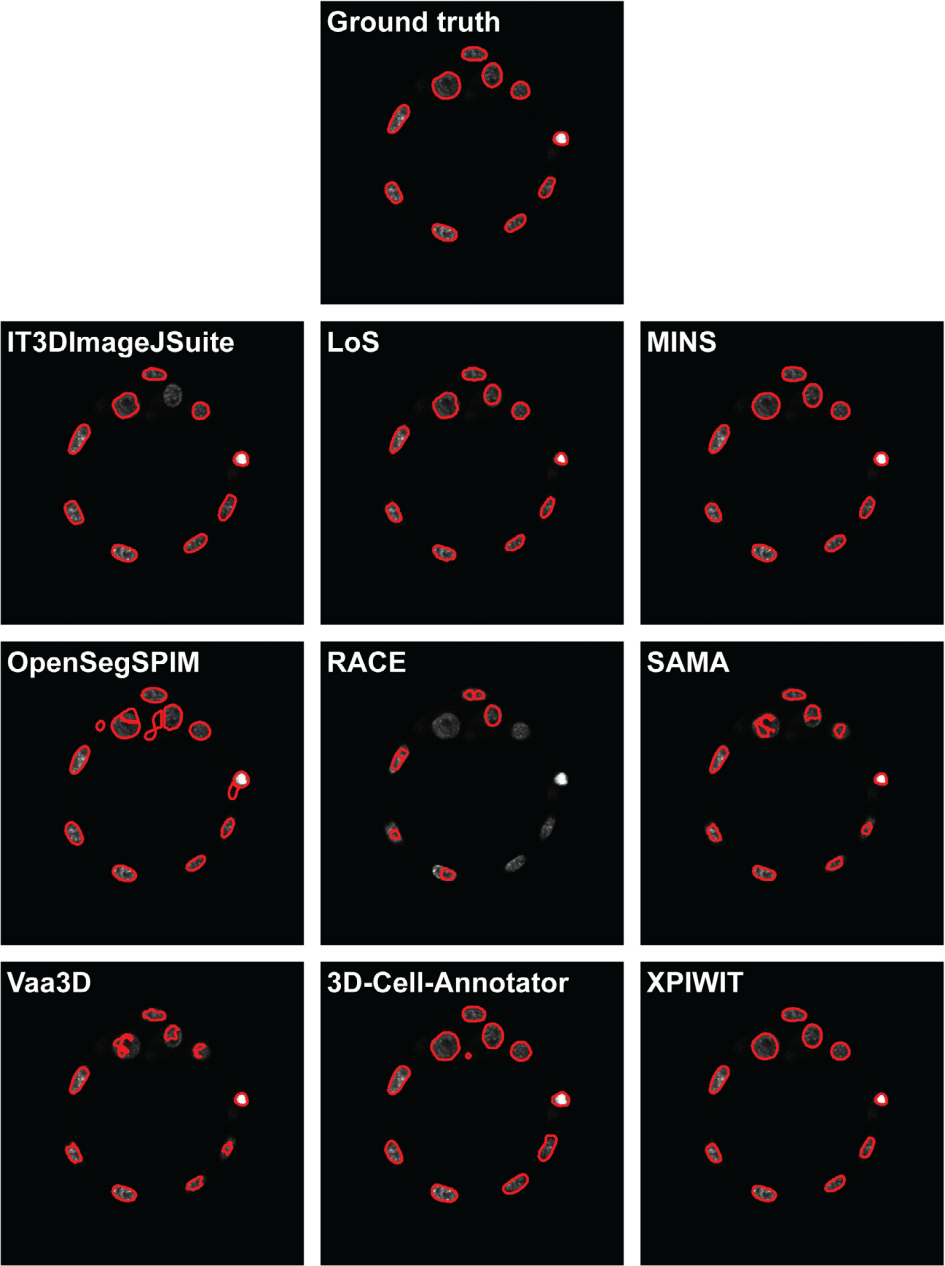
A representative section of the segmentation masks obtained on the Embryo dataset.

Fig. 2 and Fig. 3 show a representative section of the segmentation masks obtained by each tested tool on the Neurosphere and Embryo datasets, respectively. Table 3 reports the number of cells identified by each tool, and the JI values obtained. Ranking the tools and merging the two lists by assigning a penalty point for each position, has yielded the final rank reported in Table 4.

**Table 3:** Quantitative comparison of selected freely-available software tools regarding their performance in 3D segmentation of single nuclei in 3D datasets.

Tool	Neurosphere dataset			Embryo dataset		
	(detected cells)	(JI, mean ± std)	(JI mean-based rank)	(detected cells)	(JI, mean ± std)	(JI mean-based rank)
IT3DImageJSuite	23/52	0.224 ± 0.144	8	46/56	0.651 ± 0.278	4
LoS	47/52	0.398 ± 0.161	6	59/56	0.514 ± 0.230	6
MINS	48/52	0.563 ± 0.185	5	56/56	0.785 ± 0.077	2
OpenSegSPIM	51/52	0.609 ± 0.122	3	53/56	0.479 ± 0.210	8
RACE	34/52	0.393 ± 0.228	7	47/56	0.154 ± 0.141	9
SAMA	21/52	0.116 ± 0.102	9	44/56	0.485 ± 0.240	7
Vaa3D	52/52	0.597 ± 0.160	4	48/56	0.523 ± 0.342	5
3D-Cell-Annotator	51/52	0.689 ± 0.143	1	56/56	0.802 ± 0.088	1
XPIWIT	51/52	0.623 ± 0.145	2	56/56	0.742 ± 0.108	3

**Table 4:** Ranking of the tested software tools based on the quantitative comparison of their performance in 3D segmentation of single cells in 3D datasets.

Tool	Neurosphere	Embryo	Total	Final Rank
	penalty points	penalty points	penalty points	
IT3DImageJSuite	8	4	12	6
LoS	6	6	12	6
MINS	5	2	7	3
OpenSegSPIM	3	8	11	5
RACE	7	9	16	7
SAMA	9	7	16	7
Vaa3D	4	5	9	4
3D-Cell-Annotator	1	1	2	1
XPIWIT	2	3	5	2

On average, the JI values achieved for the Embryo dataset (average JI = 0.571) were better than those obtained for the Neurosphere (average JI = 0.468). This is because the Embryo dataset has higher resolution, and has a lower number of touching nuclei. Accordingly, the analyzed tools could better estimate nuclei boundaries, and were able to detect a high number of objects, which consequently scored a higher JI for the Embryo dataset. In the case of the Embryo dataset 52/56 cells were detected on average, while the same ratio was only 42/52 cells for the Neurosphere dataset (91% *vs* 81%).

Based on our analysis, the best performing tool is 3D-Cell-Annotator, which is also the most recently released one. 3D-Cell-Annotator obtained an average JI of 0.746 on our test sets. However, it is important to remark that among all the tested tools, 3D-Cell-Annotator is the only semi-automatic one, where the user defines seeding contours for the objects of interest. The highest-scoring fully-automated tool is XPIWIT, with an average JI of 0.683. However, XPIWIT requires programming experience, which can limit its use. MINS (average JI: 0.674), Vaa3D (average JI: 0.560) and OpenSegSPIM (average JI: 0.544) yielded the best segmentations among the fully-automated tools. It is important to mention that OpenSegSPIM has the advantage of providing a very handy interface to manually correct the obtained segmentations. The IT3DImageJSuite and LoS tools also reached high JI values in case of the Embryo dataset (average JI: 0.651 and 0.514, respectively), but yielded low JI values when they were utilised on the more blurry Neurosphere dataset (average JI: 0.224 and 0.398, respectively). This discrepancy strongly affected their rank positions, just followed by SAMA (average JI: 0.301) and RACE (average JI: 0.274).

## 8 7 Summary and outlook

In this work, we have extensively analysed the tools currently available for segmenting single nuclei in 3D cultures. We have divided the tools into four main categories: (*a*) tools freely available to segment 3D images (i.e. z-stacks) of nuclear stained cells; (*b*) tools freely available, but not working directly on 3D images of nuclear stained cells; (*c*) commercial tools capable of segmenting single cells in a 3D culture; (*d*) general purpose image analysis software suites providing 3D segmentation opportunities.

After briefly describing 26 tools, we have reported the quantitative comparison of 9 of them freely available for segmenting 3D images of nuclear stained cells. The comparative quantitative testing was performed on two representative datasets, comprising (*a*) a multicellular cancer spheroid imaged with an LSFM, and (*b*) a mouse embryo imaged with a confocal microscope. Providing a more extensive ranking of all available tools is out of the scope of our work, as such an aim necessitates a wider testbed. However, the short description of each tool supplemented with the JI values we achieved may give a better insight into the available software tools. This qualitative and quantitative comparison highlights their capabilities and clarifies realistic expectations regarding their use. We believe this work can be considered as the reference for researchers in the 3D field in selecting the appropriate tool for image-based single-cell analysis.

It is worth noting that deep learning solutions for segmenting single-cells in 3D are also appearing. The segmentation quality of these models strongly depend on the size and accuracy of the training dataset. No reliable quantitative test could be carried out for these tools because a sufficiently large training set was not available for us. Accordingly, we have decided to review these deep learning tools without providing quantitative results, in order not to disparage their capabilities due to the lack of sufficient training data.

All the tools considered in this work were downloaded and tested in January 2020. The specific version of each software tool used is reported in Table 1. The datasets used in the quantitative comparison test are publicly available, and the MATLAB code to compute the JI is provided at: www.3d-cell-annotator.org/download.html (Supplementary Material 1 file - “SM1”). All the masks obtained by testing the different tools are available at: www.3d-cell-annotator.org/download.html (Supplementary Material 2 file - “SM2”). Accordingly, once any new tool is developed for segmenting cells in 3D, the authors could easily compare its performance to currently existing tools by simply exploiting the test environment we have designed.

## CRediT authorship contribution statement

**Filippo Piccinini:** Conceptualization, Data curation, Formal analysis, Investigation, Methodology, Project administration, Resources, Software, Validation, Visualization, Writing - original draft. **Tamas Balassa:** Data curation, Formal analysis, Funding acquisition, Methodology, Software. **Antonella Carbonaro:** Data curation, Formal analysis, Methodology, Software, Writing - original draft. **Akos Diosdi:** Investigation, Methodology, Resources, Validation, Writing - original draft. **Timea Toth:** Data curation, Formal analysis, Methodology, Software, Writing - original draft. **Nikita Moshkov:** Data curation, Formal analysis, Methodology, Writing - review & editing. **Ervin A. Tasnadi:** Data curation, Formal analysis, Methodology, Software, Writing - original draft. **Peter Horvath:** Conceptualization, Formal analysis, Funding acquisition, Investigation, Methodology, Project administration, Resources, Software, Supervision, Writing - review & editing.
