# Supplementary figures and images for: Software tools for 3D nuclei segmentation and quantitative analysis in multicellular aggregates
Source: Comput Struct Biotechnol J. 2020 Jun 3;18:1287–300. doi: 10.1016/j.csbj.2020.05.022 (PMC7303562; doi:10.1016/j.csbj.2020.05.022)

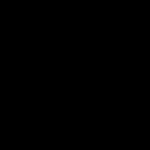

Supplement: Supplementary Data 1 [file mmc1.zip › JaccardIndex3D_JI3D/TestDataset01/Neurosphere/Spheroid_GroundTruth.tif]

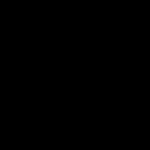

Supplement: Supplementary Data 1 [file mmc1.zip › JaccardIndex3D_JI3D/TestDataset01/Neurosphere/Spheroid_Segmentation1.tif]

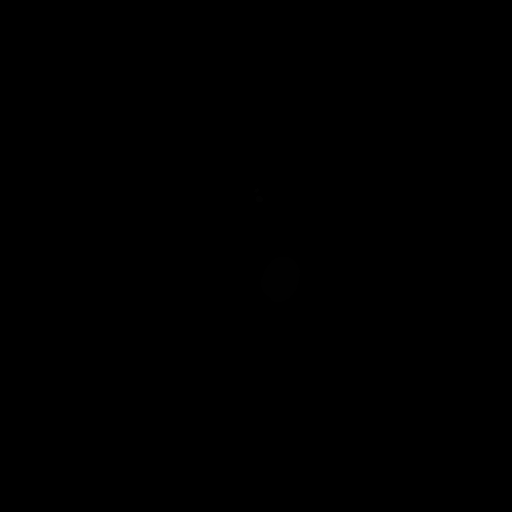

Supplement: Supplementary Data 2 [file mmc2.zip › Review_Binary3DMasks/Embryo/Embryo_3DCellAnnotator.tif]

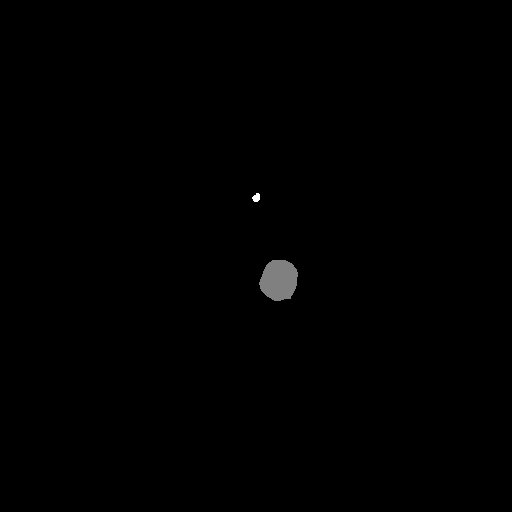

Supplement: Supplementary Data 2 [file mmc2.zip › Review_Binary3DMasks/Embryo/Embryo_GroundTruth.tif]

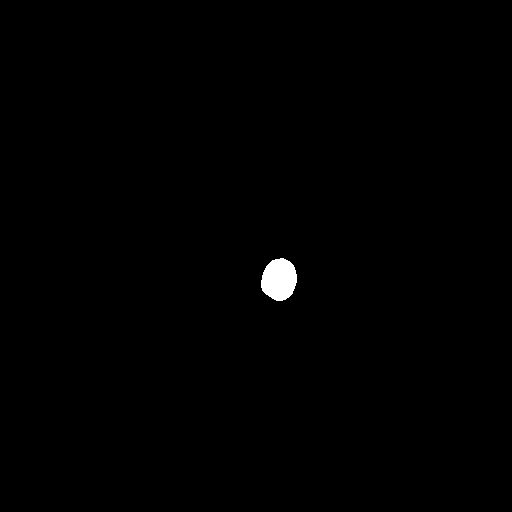

Supplement: Supplementary Data 2 [file mmc2.zip › Review_Binary3DMasks/Embryo/Embryo_IF3DImageJSuite.tif]

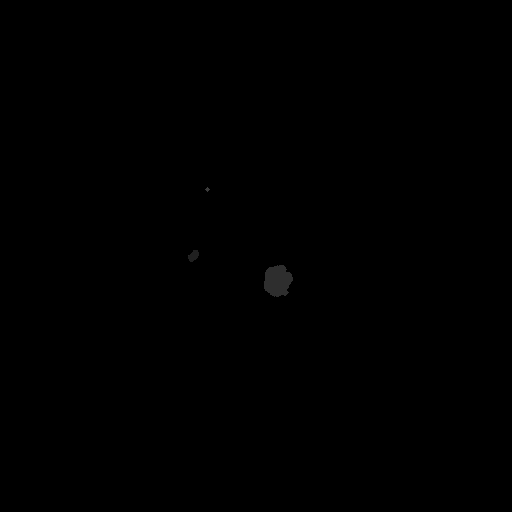

Supplement: Supplementary Data 2 [file mmc2.zip › Review_Binary3DMasks/Embryo/Embryo_LoS.tif]

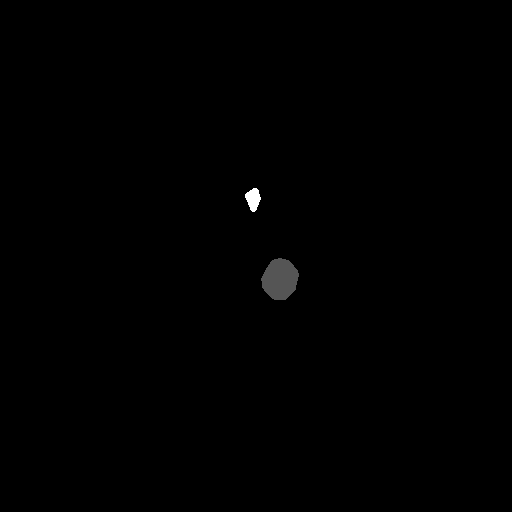

Supplement: Supplementary Data 2 [file mmc2.zip › Review_Binary3DMasks/Embryo/Embryo_MINS.tiff]

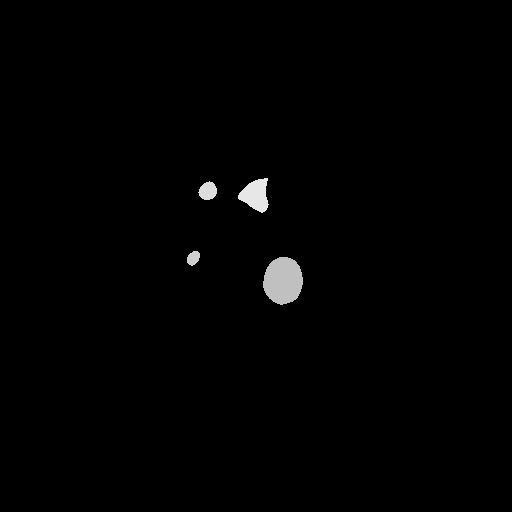

Supplement: Supplementary Data 2 [file mmc2.zip › Review_Binary3DMasks/Embryo/Embryo_OpenSegSPIM.tif]

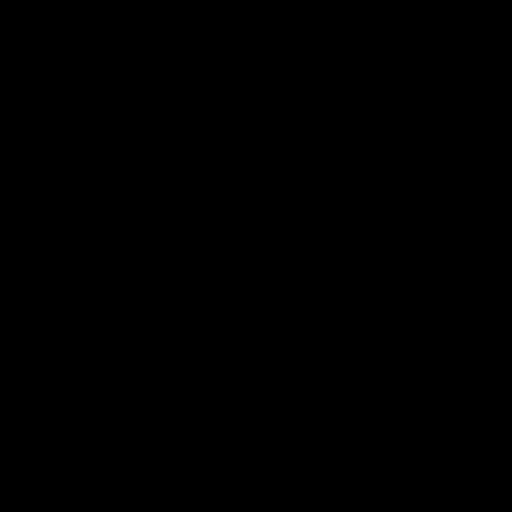

Supplement: Supplementary Data 2 [file mmc2.zip › Review_Binary3DMasks/Embryo/Embryo_RACE.tif]

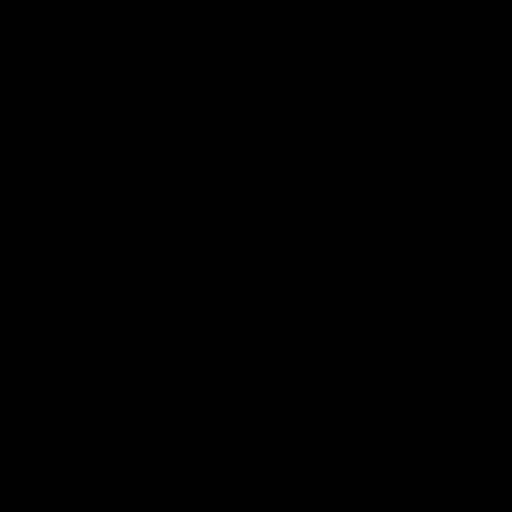

Supplement: Supplementary Data 2 [file mmc2.zip › Review_Binary3DMasks/Embryo/Embryo_Vaa3D.tif]

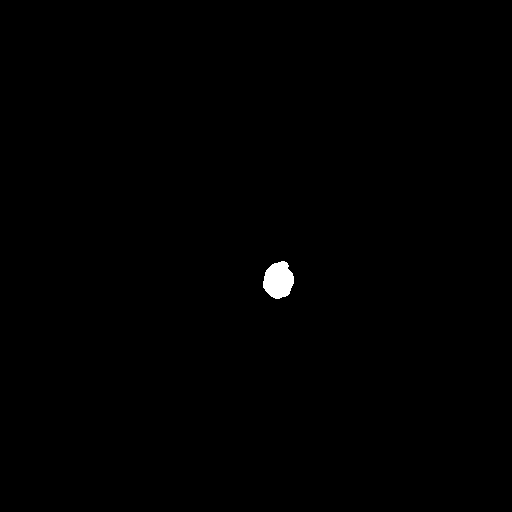

Supplement: Supplementary Data 2 [file mmc2.zip › Review_Binary3DMasks/Embryo/Embryo_XPIWIT.tif]

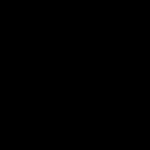

Supplement: Supplementary Data 2 [file mmc2.zip › Review_Binary3DMasks/Neurosphere/Neurosphere_IF3DImageJSuite.tif]

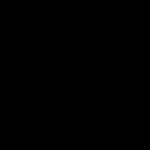

Supplement: Supplementary Data 2 [file mmc2.zip › Review_Binary3DMasks/Neurosphere/Neurosphere_LoS.tif]

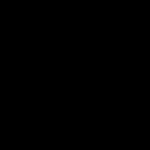

Supplement: Supplementary Data 2 [file mmc2.zip › Review_Binary3DMasks/Neurosphere/Neurosphere_MINS.tif]

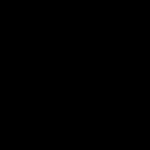

Supplement: Supplementary Data 2 [file mmc2.zip › Review_Binary3DMasks/Neurosphere/Neurosphere_OpenSegSPIM.tif]

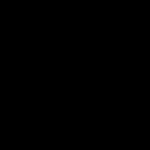

Supplement: Supplementary Data 2 [file mmc2.zip › Review_Binary3DMasks/Neurosphere/Neurosphere_Vaa3D.tif]

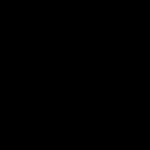

Supplement: Supplementary Data 2 [file mmc2.zip › Review_Binary3DMasks/Neurosphere/Neurosphere_XPIWIT.tif]
